# Ghosts in the machinery: Living with and beyond radiotherapy treatment for gynaecological cancer

**DOI:** 10.1177/13634593221114749

**Published:** 2022-07-28

**Authors:** Hilary Stewart, Lisa Ashmore, Mette Kragh-Furbo, Vicky Singleton, Daniel Hutton

**Affiliations:** Lancaster University, UK; The Christie NHS Foundation Trust, UK

**Keywords:** cancer, haunting, radiotherapy, treatment effects

## Abstract

This paper explores post-treatment experiences of women who have had radiotherapy for gynaecological cancer. Drawing on data from a project which explored post-treatment wellbeing, conceptual metaphors of ghosts/haunting are used to engage with enduring *legacies* of cancer and ‘neglected matters’ in post-treatment trajectories. Current arrangements of care contribute to the idea that participants are ‘out of the other side of cancer’ once active treatment completes. Despite broader ambitions for holistic cancer rehabilitation, fragilities of body and mind persist, even when the outward representation is one of health, of looking well, of moving on. We show how neglected matters of cancer (visceral late effects, psychological suffering and lives not lived) are part of living with and beyond cancer. These ‘ghosts’ manifest in chronic states of unsettledness that are temporarily relieved by individualised ‘fixes’, such as mobilisation of ‘mind over matter’ discourse and mindfulness. This discourse and its associated tools are a powerful yet impoverished framing of approaches to living with and beyond cancer. We argue for the need to attend to ‘neglected matters’ of post-treatment trajectories differently.

## Introduction

A significant amount of research exists on patients’ illness experiences of cancer. This includes research that conceptualises living with and beyond cancer as a form of ‘biographical disruption’ (Reeve et al., 2010; Trusson et al., 2016); ‘existential’ crisis (Kenne Sarenmalm et al., 2009; Röing et al., 2009); as stigmatising (Moffatt and Noble, 2015; Solbraekke and Lorem, 2016; Trusson and Pilnick, 2017); marginalising (Quincey et al., 2016); and as a ‘suffering’ (Arman et al., 2003; Sidenius et al., 2019). The framings of ‘cancer survivorship’ (Bell, 2014) and ‘coping with’ cancer (Geyer et al., 2015; Harrop et al., 2017; Navon and Morag, 2003) are also frequently used to analyse patients’ experiences of cancer. Earlier diagnosis coupled with treatment advances now means that cancer can be considered a chronic condition to be *lived with* for many more people. Holistic support has not kept pace with improved survival outcomes, making it essential to consider what life after treatment may look and feel like for the person in terms that go beyond conventional medicalised outcomes, so that we may provide appropriate support (Adams et al., 2014).

Survivorship research suggests that the desire to return to normality is a common theme amongst those living with and beyond cancer, but regaining a sense of ‘normal life’ can be difficult, and gaps in long-term support make this more challenging (Baker et al., 2016; Bilodeau et al., 2019; Trusson et al., 2016). For some, living with consequences of radiotherapy for gynaecological cancer brings physical, psychosocial and sexual challenges, as well as lingering fears about recurrence (Sekse et al., 2019). Prevalent constructions of survivorship can feel alienating, especially when these are accompanied by expectations to *move on* and put negative feelings behind them despite the ‘ongoing presence of cancer’ in their lives (Rees, 2018: 5).

We consider post-treatment experiences of cancer through the concepts of *ghosts* and *haunting.* Overend (2014) notes that concepts of ghosts/haunting have not been extensively applied to sociological understandings of illness despite their popular use in social sciences and humanities more generally. Exceptions to this include Broom et al. (2018) who analyses the *temporally* disjointed nature of living with incurable cancer, and Overend (2014: 63) who explores the *vague* nature of undefined illness (candida). Our own use of the concepts of ghosts and haunting attempts to draw together the work of Gordon (2008) with feminist work on care to attend to *neglected matters* in post-treatment cancer experiences (de la Bellacasa, 2017; Murphy, 2015). In bringing these together, we elaborate a methodological approach to engaging with and interpreting data on post-treatment experiences and needs that are neglected and made invisible by hegemonic arrangements of care, discourse and practice.

We argue that the use of ghosts and haunting in the context of post-treatment cancer experiences affords an analysis at a number of entangled levels, individual and structural. The language of ghosts and haunting provides keen metaphors for what it can be like to live with and beyond cancer: to endure side effects and vague and undefined symptoms; to live in liminal states of uncertainty, between the successions of scans, monitoring and tests (watching, waiting, anticipating/hoping/praying); to be gripped by (and keep secret) intrusive thoughts, especially those of recurrence, death and dying; to grapple with (and silence) ‘inner demons’ and mournful existential loss (of self, of direction, of life plans and of futures) (Broom et al., 2018; Hvidt, 2015; Overend, 2014; Pietilä et al., 2018).

Moreover, the critical vocabulary of the ghost summons together seemingly individual experiences into what might be described as a ‘structure of feeling’ (Raymond Williams 1977 cited in Gordon, 2008) in which affective patterns begin to emerge, knitting together across individual experiences to produce a structural haunting (Gordon, 2008). The ghost, taken together with haunting (you cannot have one without the other), thereby extends the dual meaning of the metaphor to consider structural forces and factors (Gordon, 2008), that is, socio-cultural arrangements, practices and institutions which shape experiences of cancer and reproduce/sustain the status quo of infrastructures of support. In this respect, haunting may describe or give shape to less visible or tangible forces and forms of power which shape cancer experiences. For example, the absence of holistic support may have unintended effects that manifest elsewhere in feelings of abandonment/distress or in unmet needs. The unsettling affective legacies that cancer and its treatment produce tell us that despite attempts to ‘move on’, there are unresolved matters that linger in the background which require collective reworking.

## Ghosts, hauntings and neglected matters

In her book, Ghostly Matters, Gordon (2008) attends to enduring legacies of transatlantic slavery, racial capitalism and state violence, using ghostly metaphors to trace how such unresolved and repressed injustices continue to exert a force, long after they are thought of as *over and done with*. Gordon (2008) suggests that the ghost and language of haunting bring into focus the neglected matters which reside in the background as a ‘seething presence’, notifying us of these unsettled and unresolved issues, telling us that a haunting is taking place. Therefore, things which may initially appear to belong to the past may instead represent a trouble that is being concealed or suppressed and which requires reworking.

In this respect, we suggest that the ghost and the affective urgency it brings, with its demand that things be done *otherwise*, aligns with affective ethico-political commitments of feminist *matters of care* (de la Bellacasa, 2017), meaning to *care* about neglected matters in ethical and politically committed ways. In attending to neglected matters as feminist *matters of care*, we engage with the generative *unsettling* potential (Murphy, 2015) of the ghost to trouble and interrogate hegemonic arrangements (of care, discourse and practice) and voices and experiences that have been invisibilised or silenced as a result (de la Bellacasa, 2017; Gordon, 2008). The ghost concept as we use it, therefore enables an ‘undoing and troubling of particular arrangements so that they might be acknowledged and remade in better, less violent, more liveable ways’ (Murphy, 2015: 722). While ghosts might be said to represent the legacy of a dysfunctional, sometimes harmful interaction between persons and current arrangements of care, we also suggest that learning to live *with* ghostly matters may constitute an important part of adapting to living with and beyond cancer. To engage productively with ghosts in living with and beyond cancer is then to acknowledge their presence, to negotiate with what can be seen and what lingers in the shadows (Gordon, 2008), and not least, to imagine care differently.

An integral part of how the neglected matters of the ghost come to be concealed, suppressed or dismissed as matters of the past are through hegemonic mechanisms which produce certain ways of seeing or knowing a phenomenon. Gordon (2008: 17) refers to these as dialectics of (in)visibility. For example, dominant discourses of being a cancer survivor have a particular kind of visibility (such that survivors are positioned in heroic and grateful terms or as triumphant in the face of adversity) and this neglects or invisibilises the lived experience and long-term impact of life with cancer. Such mechanisms come to foreground things which matter, whilst simultaneously suppressing neglected matters. These mechanisms are therefore important parts of a system of socio-political visibility: ‘Visibility is a complex system of permission and prohibition, of presence and absence, punctuated alternately by apparitions and hysterical blindness’ (Kipnis 1988 cited in Gordon, 2008: 16).

To make visible the neglected matters embodied in the ghost of living with and beyond cancer, we articulate findings from the data through the analytical lens of ‘technologies of (in)visibility’ to demonstrate dialectics of (in)visibility and how these work to amplify or silence experiences. Before going on to discuss our findings, below is a background on cancer treatment in the UK followed by discussion of methods used.

## Background: Cancer treatment/rehabilitation

Radiotherapy treatment for gynaecological cancer places an exceptional burden on patients. Side effects can include psychosocial and physical symptoms including depression, anxiety, fear of dying, fatigue, pain, bladder dysfunction and irritation, inflammation of the rectum, narrowing of the vaginal opening, weakening of the vaginal walls, infertility and premature menopause (Grigsby et al., 1995). Despite the significant impact of side effects on patients, the language of ‘side’ effect, as something secondary or inconsequential, is part of structuring illness experience for many patients (Digiacomo, 1989). Within literatures on gynaecological cancer experiences, patients’ *experiences* of side effects have received less explicit attention than the physiology or management of such changes. Existing research has found side effects to be experienced as ‘burdens’, particularly made worse when social support is perceived to be decreased (Schnur et al., 2009); as a ‘loss of adulthood’ in relation to issues of dignity, privacy, independence, social mobility, employability and sexuality (Rozmovits and Ziebland, 2004); and as impacting on self-identity (Chapple and Ziebland, 2002). Adams et al. (2014) suggest that as much as 50% of patients treated with pelvic radiotherapy may live with long-term gastrointestinal effects, and that 20%–50% of gynaecological cancer patients live with bowel, bladder and genitalia symptoms with significant effects on qualities of life and psychological wellbeing. The Pelvic Radiation Disease Association (Pelvic Radiation Disease Association [PRDA], 2021) estimates that in the UK, at least 100,000 people are living with long-term effects from pelvic radiotherapy. Little is known about how these patients cope in the longer-term, and studies to understand wellbeing and unmet needs of these patients are necessary to shape future interventions (Adams et al., 2014).

Responsibility for caring for patients post-treatment is unclear. Specialist cancer centres, viewed as ‘*treatment* centres’, position aftercare and support as distinct and separate to technological and other medical treatment. This is despite broader discourses surrounding personalised care and *recovery* which portrays cancer treatment in terms of more holistic rehabilitation that would include a range of services from occupational therapists and physiotherapists, dietitians and lymphoedema therapists and others. However, provision of late-effects services is patchy (Adams et al., 2014) and as we will show, post-treatment holistic support is lacking. Mental and physical health are inextricably entwined yet continue to be artificially separated by current structures and dominant arrangements of care, propagating an unsustainable mind-body dualism.

## Method

The aim of this project was to co-design a prototype digital intervention to support post-treatment wellbeing for patients who had had radiotherapy for gynaecological cancer. We adopted a multi-disciplinary and co-creation approach to meet the project aim, informed by the NIHR standards for public involvement (National Institute for Health Research (NIHR) (2018)). Four workshops were held in July and August 2019 with a range of health care professionals involved in care of patients receiving treatment for gynaecological cancer (*n* = 5) and individuals who have had previous radiotherapy treatment for gynaecological cancer (*n* = 5). The first workshop was held with staff members to establish a deep understanding of support available and the treatment pathway. This was followed by a patient only workshop to establish key areas for supporting patients on that pathway. The final two workshops were mixed staff and patients, to develop the design brief for the intervention and a review of a first response to that brief.

A purposive approach to staff recruitment was adopted to secure multi-professional representation and ensure the voices of key stakeholder groups were included in the co-creation process. Staff members included two Therapeutic Radiographers with responsibility for on treatment review, one Macmillan Support, one Specialist Trainee in Oncology and one Gynae Cancer Nurse Specialist. Patients were recruited from follow up clinics based on the following criteria: previous diagnosis of gynaecological cancer and received radiotherapy treatment for gynaecological cancer that completed more than 6 months from the date of the first workshop. Written consent was obtained from all participants at the start of each workshop. Travel reimbursement was offered to all participants and a £20 high street voucher was given as a token of thanks for participation after each workshop. The research was approved by the National Health Service (NHS) West of Scotland Research Ethics Service (WoSRES) and by the Health Research Authority (HRA) and Health and Care Research Wales (HCRW), reference 19/WS/0058.

During workshops, activities and resources were used to support participants to express views and stimulate discussions (Bloor et al., 2001; Kitzinger, 1990). These included word-clouds, prompt cards, empathy maps and persona creation tools, storylines and requirements templates. Workshops were voice recorded and recordings were iteratively listened to by members of the team who identified themes of post-treatment wellbeing. A table of themes were created with detailed summaries and timestamps added from the recordings. Relevant parts of the recordings were also transcribed. Recordings and transcriptions were then thematically analysed and interpreted in dialogue with readings of salient themes and concepts in the literature of social science and medicine and the sociology of health and illness.

Limitations of the study include its sample size. The participant group was kept small to allow everyone personal attention, as participants in the workshops were expected to actively participate in exercises and discussion and to influence the design of the intervention tool (Ørngreen and Levinson, 2017). While the aim of the workshops was to co-design an intervention tool, it was only possible through exercises and discussions that prompted participants to share and reflect on their own professional and personal experiences of radiotherapy treatment for gynaecological cancer. By doing so, participants were able to identify and discuss post-treatment wellbeing needs that the design tool had to address. As a result of those processes, new research knowledge of post-treatment wellbeing emerged (Ørngreen and Levinson, 2017) that subsequently led to the development of the Gynae Cancer Narratives research project (Ashmore et al., 2022).

## Empirical section – *Technologies of (in)visibility*

In order to *make visible* the neglected matters of living with and beyond cancer, we have framed findings from workshops under thematic headings of ‘technologies of (in)visibility’ to give shape to the hegemonic arrangements which make some matters of cancer more visible whilst simultaneously supressing other matters. Although the socio-cultural structures, practices and arrangements which frame individual experiences of living with cancer are diverse and come together in innumerable ways, we suggest some prevalent *technologies of (in)visibility* that shape post-treatment care to include:

1. biomedicine and medical technology which through scans, imaging, monitoring, physical examinations, and tests, articulate cancer and post-treatment care in terms of evidence of disease
2. dominant cancer survivorship discourses which emblazon cure/curative trajectories, the *fight/war* against cancer and ‘triumphant’ archetypes of survivorship which suppress and silence the work of living with cancer
3. ‘Mind over matter’ discourse and individualised psychotherapeutic tools such as mindfulness which quieten, suppress and ‘replace’ anxious thinking/behaviours towards cancer

These technologies of (in)visibility enact a kind of censorship on how living with and beyond cancer post-treatment can be *known*, on the narrative resources available from which to make sense of cancer experiences, and on the constituents of care arrangements. That is, alternative realities, matters and *ways of knowing* about and living with and beyond cancer are invisibilised. Although organised under apparently neat categories of (in)visibility, we note that experiences portrayed under such labels are not self-contained. Rather, they tend to bleed into one another and overlap. However, we will demonstrate that technologies of (in)visibility work to sanitise ways of knowing what it is like to live with and beyond cancer, and such ways of knowing produce adherent sociotechnical orders (de la Bellacasa, 2017).

## Biomedical visibility and empirical evidence of disease

This section will argue that biomedically focussed technologies of (in)visibility and the arrangements of care and support they engender serve to frame matters of cancer in terms of *evidence of disease* and its treatment. This rendering of cancer is made highly visible through scans, monitoring, tests, tools and follow-up appointments that punctuate the lives of those living with and beyond cancer, creating a picture of ‘cancer-as-a-clinically-known-biophysical process’ (Broom et al., 2018: 686). This *acute* framing and way of seeing cancer however, becomes a way of *not seeing* (Rappert, 2015), and suppresses experiences of living with and beyond cancer, which, we argue, may continue to emerge as an unresolved and neglected matter.

*‘Once you’ve had your treatment, you ring the bell, and you go, and then you just go, ‘well where do I go now, what do I do?’ You’ve had this team of people all ﬁghting with you, sort of looking after you, and then say, ‘off you go’ Well, what do I do? . That’s scary as well’.* (Patient 1)

As the patient quote above suggests, discussions during workshops articulated that patients felt well-supported whilst attending hospital for treatment, but that support dropped off dramatically once radiotherapy completed. Although a 6-week post-treatment triage service exists, this service is to support patients experiencing *acute* effects of treatment. Staff reported there was little post-treatment support, and that much of the support available was provided by charitable organisations. Workshop discussions revealed that follow-up appointments prioritise a certain way of seeing and knowing cancer, as the empirical evidence of disease and biomedical treatment for clinical needs, at the expense of seeing and knowing cancer in ways that are more holistic and includes psychosocial and sexual needs. Both patients and staff said that follow-up appointments are primarily concerned with annual scans and check-ups and that ‘taking the lid off the can of worms’ on psychosocial issues was avoided (Staff comment). When asked how patients were supported post-treatment, discussions at the staff workshop made very clear distinctions between biomedical treatment and psychosocial treatment, emphasising the hospital as a ‘treatment centre’ (Staff comment) and that Holistic Needs Assessments represented a ‘tick-box’ exercise (Staff comment). Responsibility for aftercare and support was therefore said to fall under the remit of other healthcare professionals, although it was not entirely clear who.

The biomedical focus of appointments was reiterated in patient workshops where patients all commented on the need to ‘look for blood’ in their underwear. As one patient told us, when attending follow-up appointments she is asked how she is, however, ‘do they mean mentally or physically? all they ask is ‘has there been any bleeding?’ no, and that’s it!’ (Patient 1). This technology of visibility we would argue, frames the terms of recovery and wellbeing through the absence or presence of blood in underwear, whilst psychosocial and sexual matters remain invisible, neglecting other elements of post-treatment wellbeing. The ghost in this patient account implores us that something is missing, that current arrangements of care fail to support psychosocial wellbeing, that such arrangements are dysfunctional and that something-must-be done, asking how we might care differently.

This issue of biomedical visibility and the relational invisibility of *neglected matters* also emerged when the topic of dilators was discussed. Patients were instructed that they should use dilators for 3–5 years following treatment to prevent vaginal stenosis so that they could be medically examined in the future. As some of the patients recalled:

Patient 5: *I got told if I didn’t use it, it would close up - it didn’t! [laughs].*

Patient 2: *Yeah I was told that. . .*

Patients expressed that they thought the dilators were unnatural, painful and intrusive, yet another demand, and a reminder of their cancer. This resonates with existing literature about dilators as a ‘rehabilitative’ practice which many women are reluctant to engage with (Bakker et al., 2015; Cullen et al., 2012). These discussions prompted a patient participant to ask a staff member at the workshop why the dilators were necessary. The staff member reiterated that this was to prevent stenosis for the sake of future exams. One patient said that she had only used the dilators for 18 months rather than the advised 5 years, and that she was worried she had not used the dilators correctly, leaving her to wonder ‘maybe that’s why it’s come back, and they can’t do any operations’ (Patient 2).

Consensus was that something needed to be done to improve the topic of dilators, but patients were not sure how this could be achieved. As taboo and as dysfunctional as the topic of dilators seems to be, they do at least get routinely mentioned and distributed to patients. However, the kind of visibility created by the dilators might be said to colonise discussions about sex and sexual health more broadly. In this respect, we suggest that the dilator as a technoscientific artefact shapes knowledge about sexual anatomy as sites for future medical exams, and not of sexual pleasure or intimacy which remains largely invisible.

This invisibility of sexual matters haunts post-treatment care, and manifests, we argue, in ghostly figures elsewhere; that is, in patient accounts about sexual health and sexuality more broadly. One patient recalled the difficulty she had dealing with desquamation (peeling skin) on her genitals from radiotherapy and that she had been using petroleum jelly to try and soothe this. She later said she discovered it was the worst thing she could have been using. When she approached medical staff for a prescription cream, she said she felt embarrassed for asking when she was told about how expensive the cream was and this prevented her from ever asking for it again. She went on to tell us that she had to push through extreme pain to be able to have sex:

*‘it’s red raw at first, the pain, it goes after a bit, but I’ve got to get past that bit, it’s horrible, it’s always the same, red raw . . . I just wish I could put something on to numb it, it’s red raw, inside, not the whole time because then I just couldn’t do it, I’ve just got to get past that bit’.* (Patient 3)

This prompted the therapeutic radiographer in the workshop to suggest a newly available lubricant, advice the patient had never had before, leading to a discussion where we questioned how patients who had received radiotherapy in the past could be expected to know about these options or latest advances in aftercare. These missing conversations about sex and sexuality with cancer may manifest in lives not lived, as one patient spoke about giving up sex since her cancer had returned:

*‘but when it comes back and you’ve got a tumour up there, you don’t feel like stuff believe you me. . . because it’s so nerve-racking, I mean, I’m 72, 73, I’m not bothered now, we had a great life in the past. . . it might like knock it or something. . .’* (Patient 2)

We suggest that the attention to dilators may be suppressing other vital conversations that *must be had* if we are to support post-treatment matters of sexuality and wellbeing. Something is missing and *something-must-be-done*. Post-treatment matters of sexual practice and sexuality fall through the cracks of support and guidance, and this indicates the broader socio-structural haunting that is taking place in the absence of sexual support. However, we suggest that the ghost of these neglected matters also gestures critically towards matters of care, imploring us to think ethico-politically about how to care differently, and how we should take responsibility for the post-treatment trajectories of patients who have had radiotherapy for cancer.

## Cure and survivorship

When active treatment completed, there was a sense that patients were expected to move on to a new phase, which participants sometimes described as being ‘out of the other side of cancer’, even those that were living with recurrence. This idea that they were out of the other side of cancer produced feelings of uncertainty about where they should go for support because they were no longer seen as *cancer patients*. Some participants told us they had rang the end of treatment bell but instead of feelings of celebration, they were left with questions about what happens next. It is at this stage that patients said they felt abandoned, as support quickly disappeared and they were left to navigate or ‘muddle through’ (Patient 1) their ‘new normal’ on their own.

Research on survivorship has shown that although concepts of survivorship are evolving, dominant understandings of cancer survival often reflect an idealised notion of the *triumphant survivor* (Bell, 2014; Dyer, 2015). This archetypal figure typically embodies ‘themes of personal transformation, heroism and triumphalism’, (Dyer, 2015), utilising war-like metaphors for cancer treatment (fight/battle against cancer) and reproducing story arcs about brave and determined individuals overcoming adversity and being stronger for it. Although this survivor identity can be an empowering narrative resource for some, it alienates many others who do not identify with it (Dyer, 2015). Similarly, existing literatures have explored the prevalence of ‘positive thinking’ when *doing illness* in cancer communities and have shown how such practices are *believed* to contribute to better outcomes (recovery, survival, cure) and better quality of life (De Raeve, 1997; McGrath et al., 2006; Wilkinson and Kitzinger, 2000).

This pressure to meet normative expectations of survivors as positive and triumphant is something we repeatedly came across during workshops. Workshop discussions were mostly quite upbeat, however at times, when we scratched the surface of some of the painful or difficult things people were living with, when the ghost began to emerge so to speak, there was what felt like a pressure to ‘be positive’ as patients quickly countered with, ‘but at least we are alive’ or ‘there are others who have it worse’.

One staff member said:

*‘I have spoken to loads of patients who have had long-term problems relating to radiotherapy, maybe 15, 20 years ago, who have said to me, but my cancer is cured, so actually these are things that I will put up with. And the people who come in are the people who are struggling. . . and they’re the ones that shout the loudest, and you worry about all the others who are just quietly putting up with it’.*

The problem of living with invisible symptoms, (bowel dysfunction, aches and pains, fatigue, anxiety and early menopause) was commonly reported by all patients who spoke about being told ‘you look well’ by others, but that this disguised what they had been through and what they were living with, and placed pressure on them to resume normal activities, such as work, before they felt ready. One participant told us that on the surface she looked well but that she struggled with health anxiety. On being discharged from follow-up care, she said:

*‘They think that that’s it now, I’m fine, and there are other people who are suffering . . . so you need to pull your socks up and move on from it and get on with your life, you’ve got nothing to worry about. . .’* (Patient 1).

Some were living with recurrence, incurable cancer as well as living with long-term effects of radiotherapy. However, these were often dismissed as ‘side effects’ that the women were putting up with or finding their own coping strategies for. This issue of hidden and necessary suffering was repeated in discussions of chronic effects and lifestyle adaptations portrayed as the price to be paid for survival, as patients reiterated they were ‘just grateful to be alive’. One patient spoke about dietary changes made to manage her bowel dysfunction (she gave up alcohol, eats bland cereal every morning, does not eat spicy food or takeaways, only drinks boiled water).

Patient 2: *I do have damage to the bowel, but as I say, most times, I do, I am, I do get very nervous, and if you’re worried about something, that’s it, straight to the loo, two or three times a day, but it’s not the runs.*

Researcher: *And do you feel comfortable with those diet changes and the not drinking alcohol and no takeaways?*

Patient 2: *Yeah I do, yeah. I’m just glad to be alive.*

Patient 5: *Yeah I feel like that.*

Patient 2: *I mean, I don’t know if you watch DIY SOS, some of the poor people on that, what a life they have, stuck in a wheelchair-*

Patient 3: *Yeah, there’s always someone who has it worse.*

She later admitted that changes to her bowel function and diet had been embarrassing, affected her mood and prevented her from staying overnight at family members’ homes, a problem she dealt with for 6 years: ‘But that’s just part of what can happen to you, y’know?’ (Patient 2).

This resonates with supporting literatures which show how the *enormity* of a cancer diagnoses overshadows post-treatment experiences and prevents discourses about late-effects of cancer and/or treatment from emerging (Pertl et al., 2014). According to Pertl et al (2014), insufficient discourse regarding cancer-related fatigue may prevent patients from raising these late-effects with practitioners. Similarly, Sidenius et al. (2019) have reported how endometrial cancer patients report feeling lucky to be alive and scale their own suffering with the suffering of others. This creates a hierarchy of suffering which delegitimises hardships patients may be experiencing and makes them less inclined to ask for support, despite ongoing adverse effects (Sidenius et al., 2019).

At another workshop, a radiographer staff member shared that she tried to prepare and reassure patients who consented to radiotherapy but who did not fully understand what it was they had consented to. These patients were understandably frightened about treatment, to which Patient 2 quickly riposted, ‘but you just want to get better don’t you, so be brave’. In this respect we suggest that biomedical technologies of visibility overlap with those of cure and survivorship. They produce highly visible matters about cancer and cure and the need to eradicate disease, to the exclusion of other understandings of what it is like to live with and beyond cancer and treatment. As Broom et al. (2018) have discussed, we might talk about how this focus on ‘curative trajectories’ as well as the imagery of ‘fighting’ cancer works to silence unpalatable experiences of living with and beyond cancer. This curative approach, which is made visible and known through treatment regimes, care practices and discourse, presents a picture of cancer as either the presence or absence of disease, and dominates how patients can think about what ‘getting better’ actually looks like or what treatment and late effects it may entail. The ghost tells us there are neglected matters to attend to, that there is something-to-be done here. However, the ghost is quickly suppressed by mechanisms of visibility that say ‘but at least you’re alive’, fetishizing notions of cure and silencing experiences of living with cancer and the extra work this involves. This omnipotence attached to ‘being cured’ or ‘surviving’ as the primary matter of concern dismisses the conditions of living with cancer, and the visceral price to be paid, as *necessary suffering*. To care differently, we must make visible the reality of living with cancer in ways which enable patients to speak up and access support for living with late-effects and psychosocial challenges.

## Mind over matter

In this section we will show how post-treatment experiences of psychosocial suffering and isolation are suppressed through the employment of idiomatic expressions such as ‘mind over matter’ and individualised psychotherapeutic approaches such as mindfulness. Although these psychotherapeutic tools are employed to reduce stress and anxiety, they may inadvertently be creating neglect of important post-treatment narratives on living with and beyond cancer.

When discussing how they coped with changes to wellbeing post-treatment, patients reported that, in the absence of formally integrated psychosocial support, they attended classes provided by charitable organisations, making use of peer support groups, 1-2-1 psychological therapy and mindfulness courses as well as holistic therapies and lifestyle groups. Patients also used apps such as Headspace and requested the digital intervention we were co-designing be able to support psychosocial wellbeing. Patients were keen for there to be a space where they could ask a health professional about concerns, to allay anxiety when they were unable to access support provided by third sector organisations. Another key requirement identified for the intervention was the need for a social networking space which enabled patients to connect with and meet other patients, with patients remarking that they had never met anyone else with a gynaecological cancer diagnosis.

During discussions on wellbeing, we came across many conversational idioms about *returning to normal, getting over/on with things, keeping busy* and *moving on from* cancer, despite lingering feelings of being unsettled:

Patient 5: *It was horrible. . . I found I just wanted to get back to me normal, I was in me last year of uni so, when I got diagnosed so, I wanted to go finish me last year in uni, go back to work. . . ‘Cos I was 35 at the time, so, I was still quite like, y’know, just wanted to be-*

Patient 2: *You had your whole life ahead of you really*

Patient 5: *Yeah, just be back to normal and stuff.*

Researcher: *and when you went back was it the same? Did you feel like ‘this is where I wanted to be’? Did it feel okay?*

Patient 5: *Yeah, it just took my mind off things, it helped, like, just makes you forget doesn’t it? Sort of, just going to work.*

Staff 5: *Gives you something to focus on.*

When discussing how cancer had affected her wellbeing, one patient said ‘it was a long time before I could enjoy things. . . I felt as though I had lost who I was, and I didn’t really want to do anything’ (Patient 1). She went onto say that she forced herself into activities but that it made her ‘feel like a fraud, because I don’t feel like that inside, but I do it, I push myself to do it, and then I think to myself, I don’t really want to do this, but I do it’. As such, a great deal of coping with challenges to wellbeing represented an idea of *going through the motions*, as was reiterated by another patient who said ‘you’ve just got to put your make up on and get dressed up’ (Patient 2) as a way of facing challenges to wellbeing.

Discussions on psychosocial wellbeing identified that patients employed idiomatic phrases such as ‘mind over matter’ and ‘being mindful’ to describe ways of coping:

Patient 4: *I remember just going for a walk and saying to myself ‘I’m going to get over this, I’m going to get over this’.*

Patient 5: *It’s mindful isn’t it, you’ve got to get in that. . .frame of mind.*

And as one patient said of the importance of keeping busy:

*‘it just takes your mind off it, mind over matter, don’t think about your stomach. . . just get on with your ironing’* (Patient 2)

And similarly, on a discussion about stress and not being able to sleep she reiterated:

*‘I had to do mind over matter, so I started taking deep breaths, in-out about six times, and then, like, started playing [theme tune] on my stomach <sings theme tune>’* (Patient 2).

Although these idiomatic expressions appear relatively benign, alluding to a mastery of difficult emotions, patients identified psychosocial wellbeing as an enduring concern that the digital intervention should care for, particularly in moments of solitude. In this respect, whilst patients suggested they were coping by ‘keeping busy’ or harnessing the power of ‘mind over matter’, much of what they actually described portrayed hidden suffering and isolation. As such, we interpreted some of the reported ‘mindful’ tactics as being suggestive of a kind of *active ignoring* and downplaying of troubles.

The troubling prevalence of these ‘mindful’ strategies became especially clear in one patient account. When talking about how her cousin was living with stage 4 cancer, she described how upbeat her cousin remained and how she wished she could have ‘just a pinch’ of that attitude:

*‘[Living positively with cancer] is a really, sort of, alien concept for me, I don’t know how she does it’.* (Patient 1)

She told us she wanted to harness mindfulness to reroute all her ‘well-trodden’ dysfunctional neural pathways and that she wanted to be able to ‘completely switch them off’. She spoke about how traumatic some of the group sessions she attended could be, especially when the topic of recurrence was raised, going on to say:

*‘It was a bit different when we were doing the mindfulness, because we were all doing something to-, we weren’t talking about our-, we’re just learning to sort of relax’.* (Patient 1)

She said that she did not know how you could deal with fear of recurrence without mindfulness, but, by her own admission, was not something she had achieved. She told us mindfulness courses gave her tools to deal with things ‘but you have to practice them or they don’t work. . . I still have really bad days now, really bad days’ and that she had to ‘work really hard to bring myself up’ using the tools she had learnt.

Here we suggest that some of these mindful strategies and ‘mind over matter’ expressions reflect an *individualising* and *pathologizing* discourse that produces feelings of deficit when patients perceive themselves as failing to cope. Drawing on Brito et al. (2021), we can think of this as a ‘side effect’ of mindfulness-based interventions that works to offset ‘systematic fallibility as individual culpability’ – the individual rather than the system is to blame for their failure to cope. As technologies of visibility, ‘mind over matter’ expressions and practices consolidate an image of what constitutes acceptable or desirable ways of wellbeing – or as Nehring and Frawley (2020: 1185) comment, ‘mindfulness discourses communicate particular ideas about solutions to social problems’. Specifically, these phrases appear to position resilience as an individual trait to be mastered through self-discipline and in isolation, rather than as a state which is reflective of, cultivated and achieved in a socially mediated context, amidst relations of care. As critics of mindfulness note, rather than enhancing wellbeing, the mainstream deployment of mindful techniques may simply encourage individuals to tolerate distress as a privatised trouble, reinforcing neoliberal expectations of rationality and self-containment (Arthington, 2016). These ‘mind over matter’ mantras therefore work to conceal enduring psychosocial struggles that patients may face when adapting to living with and beyond cancer, suppressing the emergence of narratives which grapple with questions of existential threat, fear of death, suffering and isolation. And yet, despite attempts to conceal, the ghost remains.

The ghost tells us that current arrangements of care neglect matters of psychosocial wellbeing as individual and private issues. That patients prioritised the need for the digital intervention to foster connections between patients is suggestive of the need for *collective* approaches to dealing with feelings of isolation or loneliness and post-treatment issues of wellbeing, that may complement mindful approaches.

## Conclusion

In this paper, we have proposed the conceptual metaphors of ghost and haunting (Gordon 2008) as a way of engaging with, interpreting and giving shape to neglected matters in post-treatment experiences of cancer, as well as to trouble and interrogate hegemonic arrangements (of care, discourse and practice) and voices and experiences that have been invisibilised as a result. Focusing on issues of post-treatment wellbeing, we have shown how neglected matters may manifest in chronic states of unsettledness, with long-term impacts on physical and mental wellbeing. Importantly, we argue that such individual experiences of living with cancer are shaped by, and are a part of, more structurally based hauntings. That is, they are embedded in the forces of socio-cultural arrangements, practices and institutions, which may not always be visible, but that nonetheless shape arrangements of care, and produce certain ways of seeing or knowing post-treatment experiences of cancer.

We have shown how such structural hauntings work through technologies of (in)visibilities which make some matters of cancer more visible, whilst simultaneously contributing to the suppression and silencing of neglected matters. This includes biomedicine and the production of evidence of disease, an acute framing of cancer that also becomes a way of not seeing, suppressing the experience of living with and beyond cancer; dominant cancer survivorship discourses that fetishise notions of cure, and silence post-treatment struggles and suffering; and ‘mind over matter’ rhetoric and individual psychotherapeutic approaches that while meant to enhance wellbeing, may also produce feelings of deficit and isolation. The patient accounts in this paper implore us that something is missing, that current assemblages of care have failed to fully support physical and psychosocial wellbeing post-treatment.

By combining the concepts of ghost and haunting with feminist work on care, we have argued for the generative *unsettling* potential of the ghost to make visible and engage with the neglected matters of cancer treatment. This means acknowledging the presence of ghosts that will not disappear but continue to exert a force in the lives of those they affect, and to negotiate with, the exclusions, invisibilities and concealed voices in matters of care. There are alternative realities, matters and ways of knowing life with, and beyond, cancer that are not visible, but must be attended to and must be engaged with, in order to provide appropriate support post-treatment. This is even more critical as improved survival rates from advances in diagnosis/treatment mean more people are living with and beyond cancer. We therefore propose that conceptual metaphors of the ghost and haunting constitute a powerful mode of listening, attending and negotiating with neglected matters, which demand an ethico-political reimagining of bodies and lives as they are lived, with and beyond cancer and its treatment.
